# Dual molecular diagnosis of tricho-rhino-phalangeal syndrome type I and Okur-Chung neurodevelopmental syndrome in one Chinese patient: a case report

**DOI:** 10.1186/s12881-020-01096-w

**Published:** 2020-08-03

**Authors:** Shanshan Xu, Qun Lian, Jinzhun Wu, Lingli Li, Jia Song

**Affiliations:** 1 Department of Pediatrics, The First Affiliated Hospital of Xiamen University, No.55 Zhenhai Road, Xiamen, 316003 Fujian Province China; 2 Pediatric Key Laboratory of Xiamen, No.55 Zhenhai Road, Xiamen, 361003 China; 3 Institute of Pediatrics, School of Medicine, Xiamen University, No.55 Zhenhai Road, Xiamen, 361003 China

**Keywords:** *CSNK2A1*, *TRPS1*, Tricho-rhino-phalangeal syndrome type I, Okur-Chung neurodevelopmental syndrome, Dual molecular diagnosis, Case report

## Abstract

**Background:**

Okur-Chung neurodevelopmental syndrome (OCNDS) and tricho-rhino-phalangeal syndrome type I (TRPSI) are rare Mendelian diseases. OCNDS is caused by *CSNK2A1* gene variants and TRPSI is caused by the *TRPS1*gene. However, to have two Mendelian diseases in one patient is even rarer.

**Case presentation:**

A 6-year-10-month-old boy characterized by special facial features, short stature and mental retardation was referred to our pediatric endocrinology department. Whole-exome sequencing (WES) was done to detect the molecular basis of his disease. This patient was confirmed to carry two variants in the *CSNK2A1* gene and one in the *TRPS1* gene. The variant in the *CSNK2A1* gene was vertically transmitted from his father, and the variant in *TRPS1* gene from his mother. These two variants are classified as pathogenic and the causes of the presentation in this child. This patient’s father and mother have subsequently been diagnosed as having OCNDS and TRPSI respectively.

**Conclusion:**

This is the first reported case of a dual molecular diagnosis of tricho-rhino-phalangeal syndrome type I and Okur-Chung neurodevelopmental syndrome in the same patient. This patient is the first published example of vertical transmission of this recurrent *CSN2A1* variant from parent to child. A novel variant in the *TRPS1* gene that is pathogenic was also identified. In conclusion, identification of the variants in this patient expands the phenotypes and molecular basis of dual Mendelian diseases.

## Background

The tricho-rhino-phalangeal syndrome (TRPS) was first described by Andres Giedion in 1966 [1]. It is a rare autosomal dominant disorder characterized by distinctive facial features and skeletal abnormalities, such as sparse scalp hair, a peculiar shaped nose, cone-shaped epiphyses of the phalanges, and short stature [2]. TRPS comprises three subtypes, TRPS I (OMIM #190350), TRPS II (OMIM #150230) and TRPS III (OMIM #190351) depending on clinical presentation and molecular differences. TRPS I and TRPS III are caused by heterozygous mutations of the *TRPS1*gene. TRPS III differs from TRPSI by the presence of severe brachydactyly due to short metacarpals and severe short stature [3]. TRPS II is caused by a contiguous gene deletion of *TRPS1, RAD21*, and *EXT1* with multiple osteochondromas, which is absent in TRPS I patients [4, 5]. *TRPS1* gene encode a zinc-finger, GATA-type transcription factor, which is involved in the development and differentiation of the bones, kidneys, and hair follicles [2].

Okur–Chung neurodevelopmental syndrome (OCNDS, OMIM #617062) is an autosomal-dominant disorder which is characterized by intellectual disability, developmental delay, behavioral problems, and other multisystemic abnormalities. It is caused by *CSNK2A1* gene variants which was first reported by Okur in 2016 [6]. *CSNK2A1* gene is expressed in the brain and encodes the catalytic subunit of protein kinase CK2, which is in involved in many biologic processes.

Usually, it is hard to diagnose these rare Mendelian diseases through routine clinical practice such as the recognition of phenotypes, biochemical tests and imaging. Whole-exome sequencing (WES) provides a way to reveal the relationship between clinical phenotypes and genotypes. Recent studies show that 4.9% (101/2076) of patients have had more than one molecular diagnosis [7]. In this case study, a 6-year-10-month-old boy characterized with special facial features, short stature and mental retardation was confirmed to be carrying compound variants in both the *CSNK2A1* gene and also the *TRPS1* gene using WES. This patient has a dual molecular diagnosis of tricho-rhino-phalangeal syndrome type I and Okur-Chung neurodevelopmental syndrome.

## Case presentation

### Patient and clinical evaluation

A 6-year-10-month-old boy was referred to the pediatric endocrinology department with short stature and developmental delay. This boy was the first child of non-consanguineous parents. He was born at full term. His birth length was 48 cm (− 1.33 s.d.) and birth weight was 2.65 kg (− 1.80 s.d.). His mother noted he had poor growth after the age of 1, but didn’t know the exact growth velocity. His developmental milestones were delayed. He first walked by himself and spoke at 4 years of age. He also suffered from constipation. At 6 years, he started primary school, but struggled to socially adapt to his peer group. He was unable to express phrases and communicate with other children of his age. His father is 147 cm (− 4.01 s.d.) tall, with unusual facial features such as a round face, broad nasal bridge, short upturned nose and arched eyebrows. He has mild intellectual disability. Paternal grandparents’ heights are 160 cm and 150 cm, both are of normal intelligence. The patient’s mother is 141 cm (− 3.63 s.d.) tall, with facial features such as sparse scalp hair and a pear-shaped nose. She is of normal intelligence. However, maternal grandparents’ details are unknown due to adoption.

**Fig. 1:**
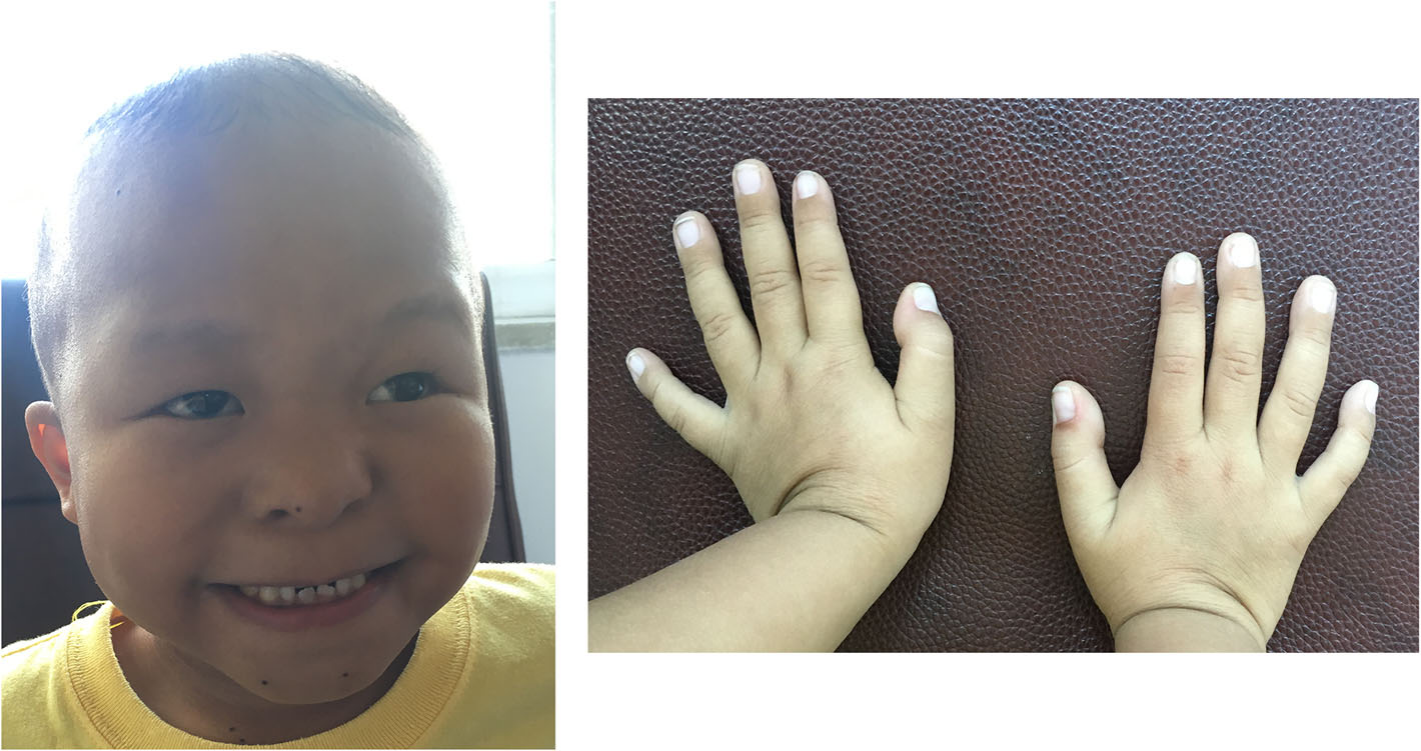
Distinctive face and hands of the patient in this study. Facial dysmorphic features include sparse scalp hair, a pear-shaped nose, long flat philtrum and thin upper vermillion border

At the time of presentation at 6 years 10 months, his height was 94.5 cm (− 5.8 s.d.), his weight was 12.8 kg (− 4.3 s.d.), and his head circumference was 50.1 cm (− 1.0 s.d.). Physical examination showed facial dysmorphic features such as sparse scalp hair, a pear-shaped nose, long flat philtrum, thin upper vermillion border and short stature (Fig. 1). Standard blood and urine screening tests were normal, and a growth hormone (GH) provocation test with arginine/levodopa showed appropriate GH peaks. No disturbances of calcium/phosphate metabolism were found. The bone age, performed according to the atlas of Greulich and Pyle was significantly delayed (bone age 2 years at 6 years and 10 months chronological age). Echocardiography was normal. MRI of the brain and pituitary region revealed a reduced size anterior pituitary gland. On the Wechsler Intelligence Scale for Children (Chinese Revision) (WISC-RC) he was found to perform at the level of severe intellectual disability (Intelligent Quotient < 40).

His parents have given informed consent for his pictures and medical data to be published.

### WES analysis and sanger sequencing

Peripheral blood samples were collected from the patient and his parents after obtaining written informed consent. Single WES was done on this patient’s blood, then Sanger sequencing was performed on his parents’ blood samples to verify the variants. The genomic DNA was extracted from peripheral blood samples by using the QIAamp DNA Blood Mini Kit (Qiagen, Hilden, Germany) according to the manufacturer’s instructions. 1.5 μg genomic DNA was fragmented into an average size of 300 bp, then the fragmented genomic DNA was used for preparation of sequencing libraries. 8 bp barcoded sequencing adaptors were then ligated with DNA fragments before final hybridization with xGen® Exome Research Panel v1.0 focused exon probes (IDT, USA). All the exons were sequenced by Illumina HiSeq X-10 platform. Purified sequencing libraries were pooled together and massively parallel sequenced by Illumina HiSeq X platform to produce a sequencing yield of 10.0 Gb raw data. The reads were mapped to the reference human genome using NextGene software (SoftGenetics LLC, State College, PA). All the variants were compared with databases such as 1000 Genomes Project, the Exome Aggregation Consortium (ExAC) and dbSNP. The variants with a minor allele frequency greater than 0.01 in the control databases were excluded. Pathogenic predication analysis was carried out on platforms of SIFT, Polyphen-2 and mutation taster. All the variants were studied to confirm if they were located in the conserved gene region and if the variants affected the protein function or structure. Following these filtering steps, potential variants were considered to cause disease and were subsequently validated by Sanger sequencing. The mean read depth of this patient against RefSeq protein-coding regions was 93.24 reads with 97.71% being covered by ⩾20 reads, and 93.70% covered by ⩾30 reads.

**Fig. 2:**
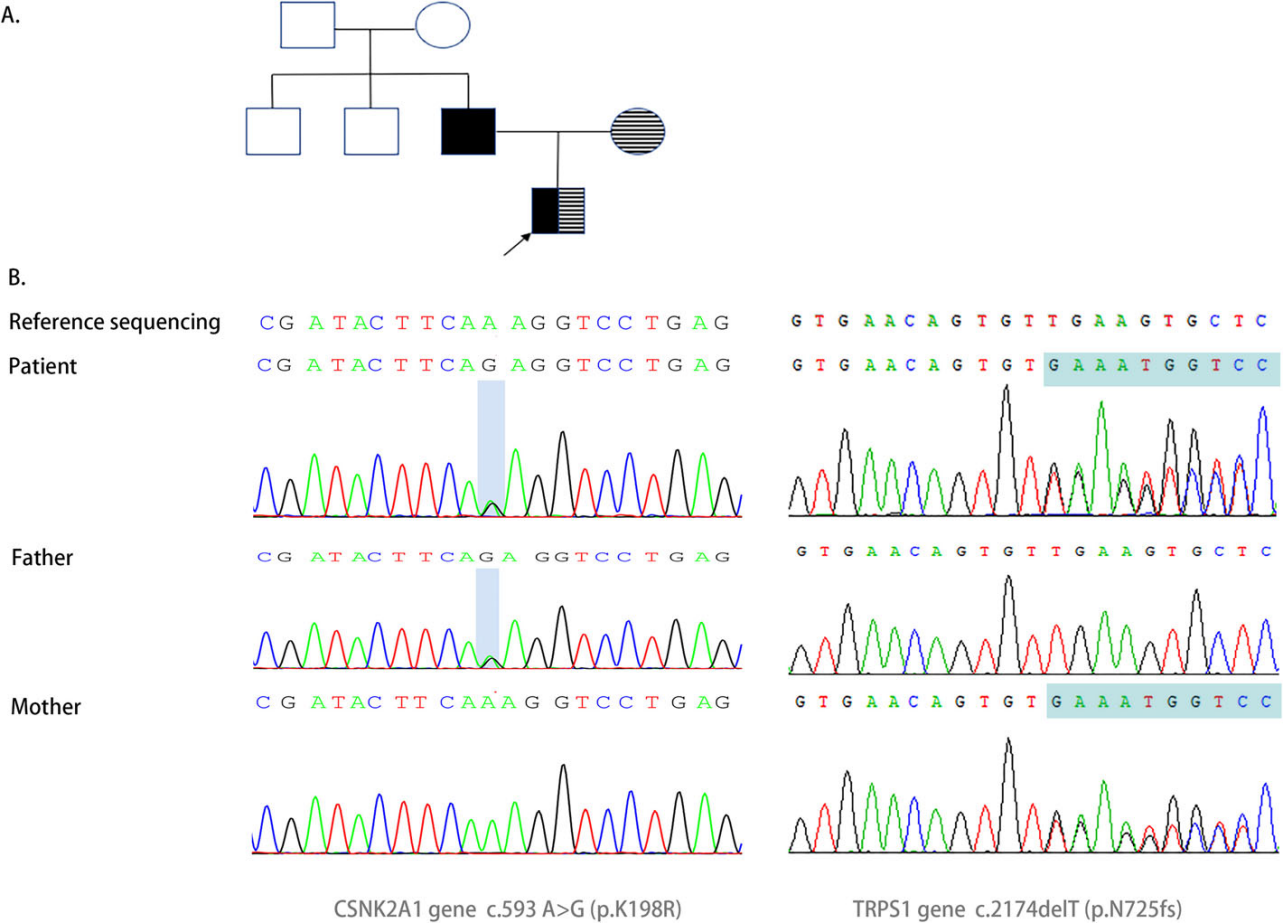
**a** Patient’s family’s pedigree. Black arrow shows the proband. Black square indicates the patient affected with OCNDS caused by a variant in the *CSNK2A1* gene. Square filled with transverse lines indicates the patient affected with *TRPS I* caused by a variant in the *TRPS1* gene. The compound heterozygote found in this patient was vertically transmitted from his father and mother respectively; **b** Validation by Sanger sequencing of *CSNK2A1* and *TRPS1* gene in this family

We identified a *CSNK2A1* variant (NM_177559: c.593 A > G, p.K198R) and a *TRPS1* variant (NM_014112:c.2174delA, p.N725fs), which were vertically transmitted from his father and mother, respectively (Fig. 2). The variant c.593 A > G(p.K198R, rs 869,312,840) on *CSNK2A1* gene has previously been reported in the medical literature as ‘pathogenic’ on at least six previous occasions (PS4-Strong) [8–11]. This variant is in the critical protein domain (PM1) and it is extremely rare in the general population (PM2). The patient’s phenotype is consistent and specific for Okur–Chung neurodevelopmental syndrome (PP4). According to American College of Medical Genetics and Genomics/The Association for Molecular Pathology (ACMG/AMP) variant interpretation guidelines [12], this variant can be classified as pathogenic. The variant c.2174delA(p.N725fs) on *TRPS1* was a novel variant, which causes a frame shift. This variant affects the amino acid residue number 725 and introduces a premature stop codon after 39 residues in the gene sequence. This is a loss of function variant (PVS1), which is absent in general population (PM2). The patient’s and his mother’s phenotypes are highly specific for TRPS I (PP4). This can be classified as pathogenic based on ACMG/AMP guidelines [12].

## Discussion and conclusions

In this report we describe a dual molecular diagnosis of OCNDS and TRPS1 in a Chinese patient. This patient has inherited both clinical presentations of OCNDS and TRPS I (Table 1), and his father and mother present typical manifestations of OCNDS and TRPS I respectively.

**Table 1:** Clinical characteristics of our patient compared with TRPS and OCNDS features

	Our patient	TRPS I^a^	OCNDS^a^
Growth/Height	Short stature (− 5.8 s.d.)	Short stature Normal birth length	Failure to thrive (in some patients)
Face, ears, eyes, nose, mouth& teeth	Prominent, long philtrum Thin upper lip Pear-shaped nose	Prominent, long philtrum Thin upper lip Pear-shaped nose Small, carious teeth Large prominent ears	Hypertelorism, Epicanthal folds, Arched eyebrows, Synophrys, Ptosis, Low-set, folded ears Broad nasal bridge, upturned nose High palate, thin upper lip
Skin, nails & hair	Sparse, thin hair Slow-growing hair Thin eyebrows	Sparse, thin hair, Slow-growing hair, Thin nails, Thin eyebrows	Not mentioned
Intellectual disability	Severe intellectual disability Delayed speech Poor speech	Normal intelligence	Global developmental delay Intellectual disability Delayed speech Poor or absent speech
Neurologic and behavior problem	Behavioral problems: Attention deficit	Hypotonia (infancy)	Hypotonia Behavioral problems: Tantrums, Volatile mood, Hand-flapping, Attention deficit-hyperactivity disorder
Skeletal	Delayed bone age	Delayed bone age before puberty, Cone-shaped epiphyses of middle and proximal phalanges (2nd, 3rd, 4th fingers)	Joint hyperextensibility (1 patient) Scoliosis (1 patient)
Cardiac	–	Not mentioned	Congenital heart defects (in some patients)
Gastrointestinal symptom	Constipation	Not mentioned	Feeding difficulties - Constipation - Gastro-esophageal reflux
Immunologic	–	Not mentioned	Hypogammaglobulinemia (in some patients) - IgA deficiency - IgG deficiency

^a^ Data comes from OMIM (https://www.omim.org)

OCNDS is a new syndrome, which was first described in 2016 by Okur et al. [6]. Common features included development delay, intellectual disability, variable dysmorphic facial features, gastrointestinal problems and musculoskeletal abnormalities. Patients may present with microcephaly, low-set and folded ears, arched eyebrows, epicanthal folds, broad nasal bridge, upturned nose, or a thin upper lip. To date, no more than 60 individuals with variants in *CSNK2A1* have been reported [8–11, 13, 14]. Most of these variants are de novo variants. The variant detected in this patient was vertically transmitted from his father, who also presents with clinical features of OCNDS. Actually, this is the first OCNDS family report. The variant c.593 A > G (p.K198R) is common in OCNDS, which was previously described in the literature in several other affected patients, so it is thought to be a “hot spot” [8–11]. Interestingly, this patient is the first published example of vertical transmission of this recurrent *CSN2A1* variant from parent to child. There is some overlap between these clinical reports and our patient, such as delayed development and intellectual disability, but not every affected individual has growth retardation as in our patient, some may actually be of normal height (above − 2.0 s.d.).

TRPS I was first described in 1966, and can be diagnosed based on clinical criteria [1]. This patient fulfill sufficient features to meet the clinical criteria for this diagnosis with the presence of sparse scalp hairs, a bulbous and pear shaped nose, long philtrum and brachydactyly. His mother had similar features. Maas SM et al. [15] described the phenotype and genotype in 103 patients with TRPS. Decreased linear growth was observed in almost all patients. It is more obviously postnatally than prenatally, and half of the adult patients are below − 2 s.d. in height. Growth hormone supplementation has been used in patients with TRPS; however the response to GH therapy is variable in affected individuals [16]. The percentage of TRPS I individuals with intellectual disability is similar to that in the general population [15], so the reason that the described patient has low IQ score could be explained by his also affected by OCNDS. Almost all patients have cone-shaped epiphyses, which can also be detected at an early age. However, our patient doesn’t show this characteristic radiological feature, consistent with the variable clinical presentation of TRPS.

Molecular methods confirmed the diagnosis of TRPS I in this patient and his mother. The *TRPS1* gene contains 7 exons, and encodes 1294 amino acids. Most of the mutations are nonsense and frameshift mutations which are located in exons 4–7 of the gene. The number of recurrent mutations is very low [15]. Variant c.2174delA(p.N725fs) is novel, located in exon 5. This alters the *TRPS1* reading frame and results in the introduction of a premature stop codon.

Co-occurrence of two Mendelian diseases in one patient is very rare. Posey et al. [7] summarized a retrospective analysis of data from 7374 patients who had been referred for whole exome sequencing. Amongst these patients, 2076 (28.2%) had at least one molecular diagnosis, but only 101 (4.9%) were diagnosed with two or more disease loci. In the same patient, the two Mendelian diseases could be distinct or overlapping by phenotype.

Our patient has inherited two variants of the *CSNK2A1* gene and the *TRPS1* gene separately from his father and mother. These two syndromes share the same presentation “short stature”. However, compared with other patients with single OCNDS or TRPS I, and with his father (− 5.8 s.d. VS − 4.01 s.d.) or his mother (− 5.8 s.d. VS − 3.63 s.d.), this patient who has dual diagnosis of OCNDS and TRPS I, has a more significant degree of short stature. These two pathogenic variants of the *CSNK2A1* and *TRPS1* genes perhaps have a combined exaggerated deleterious effect on linear growth. Whilst these two syndromes have different facial features, this patient resembles his mother.

We referral the patient for speech and language therapy. We suggested growth hormone therapy to improve short stature, but the family rejected this option for economic reasons.

In summary, our study describes a Chinese patient who suffered from simultaneous tricho-rhino-phalangeal syndrome type I and Okur-Chung neurodevelopmental syndrome. The variants in *CSNK2A1* gene were vertically transmitted from his father and a variant in *TRPS1* gene from his mother. The recurrent variant c.593 A > G(p.K198R) in the *CSNK2A1* gene has not previously been described in two members of the same family. A novel variant c.2174delA(p.N725fs) in the *TRPS1* gene that is pathogenic was also identified. Further functional analysis of this variant could expand the mechanism exploration of TRPS I. Dual Mendelian diseases in one patient is rare; however, with the availability of molecular diagnostic methodology, we may be able to identify more patients with multiple molecular diagnoses, and in turn, improve knowledge about inherited human diseases.

## Data Availability

The datasets used and analyzed during the current study are available from the corresponding author on request.

## Abbreviations

WES: Whole-exome sequencing
TRPS: Tricho-rhino-phalangeal syndrome
OCNDS: Okur–Chung neurodevelopmental syndrome
GH: Growth hormone
s.d.: Standard Deviation
